# Experimental evidence for the role of paramagnetic oxygen concentration on the decay of long-lived nuclear spin order†

**DOI:** 10.1039/c9ra03748a

**Published:** 2019-07-29

**Authors:** Bryan Erriah, Stuart J. Elliott

**Affiliations:** School of Chemistry, University of Southampton Southampton SO17 1BJ UK stuart-james.elliott@univ-lyon1.fr

## Abstract

Nuclear singlet lifetimes are often dependent on the quantity of paramagnetic oxygen species present in solution, although the extent to which quenching or removing molecular oxygen has on extending singlet lifetimes is typically an unknown factor. Here we investigate the behaviour of the singlet relaxation time constant as a function of the oxygen concentration in solution. An experimental demonstration is presented for a chemically inequivalent proton pair of the tripeptide alanine–glycine–glycine in solution. We introduce a simple methodology to ensure the solution is saturated with predetermined concentrations of oxygen gas prior to measurements of the singlet lifetime. Singlet lifetimes were measured by using the spin-lock induced crossing pulse sequence. We present a linear relationship between the amount of oxygen dissolved in solution and the singlet relaxation rate constant. Singlet relaxation was found to be ∼2.7 times less sensitive to relaxation induced by paramagnetic oxygen compared with longitudinal relaxation. The relaxation behaviour is described by using a model of correlated fluctuating fields. We additionally examine the extension of singlet lifetimes by doping solutions with the chelating agent sodium ascorbate, which scavenges oxygen radicals in solution.

## Abstract 2

The sensitivity of longitudinal magnetization and singlet order to relaxation from dissolved paramagnetic oxygen sources in solution is investigated experimentally.

## Graphical abstract

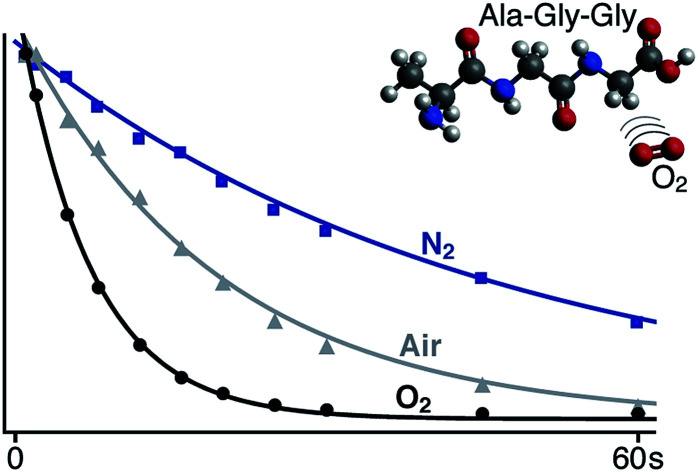

## 1 Introduction

The observation of non-equilibrium magnetization in solution is limited by the spin–lattice relaxation time constant *T*_1_. Long-lived states (LLS) are protected against the in pair dipole–dipole relaxation mechanism and decay with extended lifetimes,^1–25^ providing a means of bypassing this limitation. For spin-1/2 pairs, the long-lived state is defined as the mean population imbalance between the exchange-antisymmetric nuclear singlet state and the exchange-symmetric nuclear triplet states.^1,2^ The long-lived state is referred to as nuclear singlet order, and has a corresponding decay time constant denoted *T*_S_. LLS lifetimes dwarfing *T*_1_ by a factor of 50 have been observed,^26,27^ with a LLS lifetime exceeding 1 hour recorded for a ^13^C_2_-labelled naphthalene derivative to room temperature solution.^28^ LLS have applications to ligand-binding,^29–32^ reaction monitoring^33^ and imaging contrast.^34^ The combination of LLS with hyperpolarization techniques has also been proposed.^35–41^

Numerous relaxation mechanisms attenuate singlet lifetimes including: out-of-pair dipole–dipole couplings,^42,43^ chemical shift anisotropy,^28,43^ scalar-relaxation-of-the-second-kind,^44,45^ singlet–triplet leakage^43,46^ and spin-rotation/spin-internal-motion.^28,47^ Dissolved paramagnetic impurities in solution additionally contribute to the singlet relaxation rate constant *T*_S_^−1^. A previous study from Tayler and Levitt explored the dependence of nuclear singlet lifetimes on the concentration of transition metal and lanthanide salts present in solution.^48^ Relaxation caused by paramagnetic oxygen dissolved in solution is also an important singlet decay mechanism. In some cases, expelling dissolved oxygen from solution can dramatically lengthen the observed singlet lifetime.^26,27,49,50^ To the best of our knowledge, a detailed study regarding the behaviour of singlet relaxation times as a function of paramagnetic oxygen concentration in solution has not been reported.

**Fig. 1:**
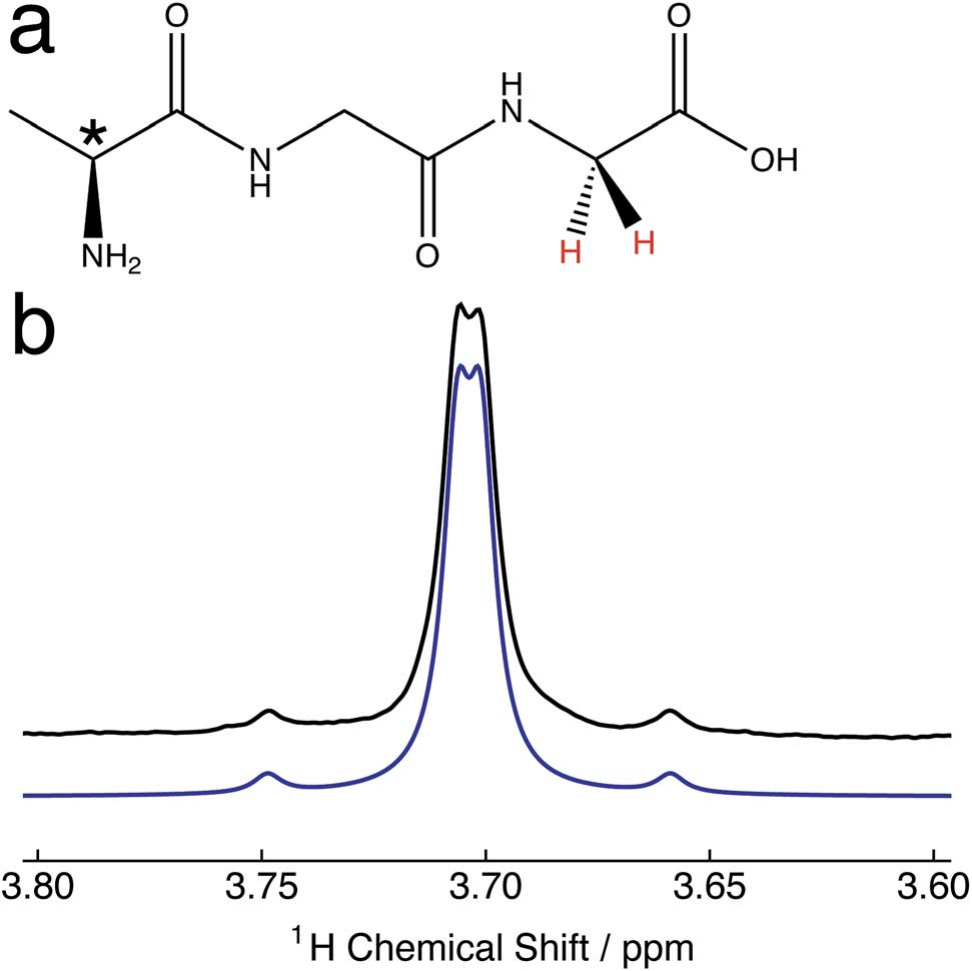
(a) Molecular structure of the polypeptide alanine–glycine–glycine (Ala–Gly–Gly). The proton pair participating in the singlet order is coloured red. * denotes the molecular chiral centre. (b) Relevant portion of the ^1^H NMR spectrum of 25 mM Ala–Gly–Gly dissolved in degassed D_2_O solution acquired at 9.4 T (^1^H nuclear Larmor frequency = 400 MHz) and 298 K with a single transient. Black line: experimental spectrum; blue line: simulated spectrum (*J*_HH_ = 16.9 Hz; Δ*ν* = 8.8 Hz), using Lorentzian line broadening (half-width at half-height = 3.0 Hz).

In this communication, we investigate the singlet lifetime behaviour for a proton pair of the polypeptide alanine–glycine–glycine (Fig. 1a) as a function of the oxygen concentration in solution. A straightforward approach to establish a desired concentration of oxygen in solution is described. Singlet and longitudinal relaxation rate constants were found to have a linear dependence on the concentration of oxygen in solution, with nuclear singlet order observed to be a factor of ∼2.7 less sensitive to paramagnetic oxygen induced relaxation compared with ordinary magnetization. A description of the relaxation behaviour using a model of correlated fluctuating fields is discussed. Singlet lifetimes are additionally measured in solutions containing the oxygen radical scavenging agent sodium ascorbate.

## 2 Methods

10.2 mg of alanine–glycine–glycine (Ala–Gly–Gly) was dissolved in 2 mL of D_2_O solvent at a concentration of 25 mM. Samples were transferred to NMR tubes with an outer diameter of 10 mm.

The relevant portion of the experimental proton NMR spectrum corresponding to the α-protons of the Gly terminal residue of Ala–Gly–Gly is shown in Fig. 1b. The two central peaks of the AB spectral pattern are resolved, and are separated by an inner splitting of 1.7 Hz. Fig. 1b indicates that the two protons are strongly-coupled, *i.e.* there is a small chemical shift difference between the two protons sites with respect to the in pair *J*-coupling. The constituent singlet nuclei are diastereotopic due to the presence of a molecular chiral centre (*, Fig. 1a). The experimental proton NMR spectrum can be well simulated using the following parameters:^51^*J*_HH_ = 16.9 ± 0.3 Hz; Δ*ν* = 8.8 ± 0.1 Hz, which are in approximate agreement with the literature.^52^

**Fig. 2:**
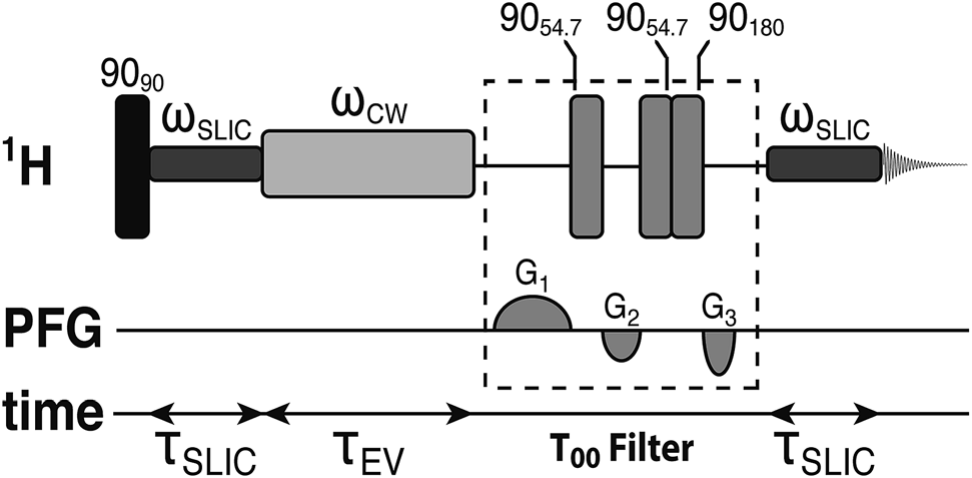
Schematic representation of the spin-lock induced crossing (SLIC) radiofrequency pulse sequence used for accessing the singlet order between the two alpha protons in the Gly terminal residue of the tripeptide Ala–Gly–Gly and measuring its decay. The experiments used the following parameters: *ω*_SLIC_/2π = *J*_HH_ = 16.9 Hz, *τ*_SLIC_ = 100 ms, *ω*_CW_/2π = 300 Hz.

The small proton chemical shift difference allows access to the long-lived nuclear singlet order, by using pulsed methods,^52–54^ and their variants,^46,55–59^ which operate efficiently in the near-chemical equivalence regime. In the current study, we employed a modified version of the spin-locking induced crossing (SLIC) pulse sequence,^54^ as shown in Fig. 2. Details of the pulse sequence optimization and the T_00_ filter are given elsewhere.^60,61^ The sequence converts the spin-locked magnetization into singlet order through the action of the chemical shift difference, with conversion complete in a time *τ*_SLIC_ ≃ 2^−1/2^Δ*ν*^−1^, where Δ*ν* is the proton chemical shift difference in hertz, neglecting relaxation and other complications. The parameters of the SLIC pulse were adjusted to achieve optimal triplet–singlet population conversion: *ω*_SLIC_/2π = *J*_HH_ = 16.9 Hz; *τ*_SLIC_ = 100 ms. The singlet order is allowed to evolve for a variable time interval *τ*_EV_ in the presence of an on resonant continuous wave (CW) radiofrequency field, with nutation frequency *ω*_CW_/2π = 300 Hz, which suppresses relaxation contributions from singlet-triplet mixing.^43^ The maximum integral of the singlet-filtered ^1^H NMR signal, relative that induced by a single 90° pulse, was found to be 0.37. The loss relative to the theoretical maximum of 2/3 is not yet fully understood but is attributed to radiofrequency field imperfections, static magnetic field inhomogeneities and relaxation.^62^ Since *J*_HH_ ∼ 2Δ*ν* the performance of the SLIC pulse may not be optimal, which would lead to additional losses in triplet–singlet population conversion.^54^

A stringent requirement of this study is the accurate determination of the concentration of molecular oxygen dissolved in solution. As such, a key sample preparation step is the exposure of the D_2_O solution to a predetermined weight percentage (wt%) of O_2_ gas (*via* sample bubbling) for a sufficient amount of time as to allow full saturation of the O_2_ gas in solution.

To achieve a satisfactory saturation of oxygen gas, a sufficient solution bubbling time was determined from a series of calibration experiments. Samples were exposed to three gasses (O_2_ gas wt% given in brackets); nitrogen (<0.01), compressed air (∼20.95) and oxygen (>99.99), for incremented time periods (up to 180 s) until no discernible changes in the measured singlet or longitudinal relaxation times were observed. J.-Young low pressure/vacuum (LPV) NMR tubes (outer diameter = 10 mm) were connected to gas cylinders *via* 1/16 inch PTFE tubing and a glass NMR pipette. The gas flow rate was 29 mL min^−1^. The dead space of the NMR tube above the solution was exposed to gas for 10 s following the solution bubbling process.

For singlet and longitudinal lifetime measurements as a function of the dissolved oxygen concentration in solution, samples were bubbled for 180 s with a range of N_2_ : O_2_ gas mixtures. Cylinders of mixed N_2_ : O_2_ gases were purchased from BOC Ltd UK. Cylinders were 1.2 L in volume and pressurized to 200 bar. The wt% of O_2_ gas ranged from 2.5%–17.5% in 2.5% increments. Errors on the wt% of O_2_ gas in each cylinder were provided by BOC Ltd UK. Singlet relaxation times *T*_S_ were estimated by using the SLIC pulse sequence described in Fig. 2. Longitudinal relaxation times *T*_1_ were measured by using separate inversion-recovery experiments.

## 3 Results

**Fig. 3:**
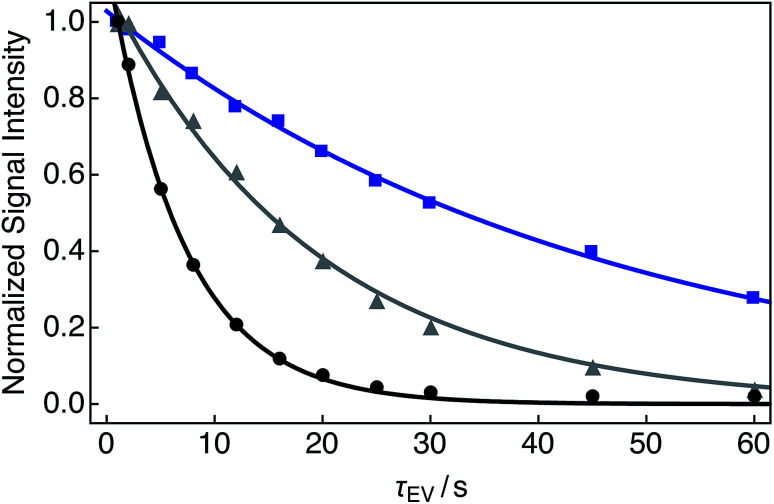
Experimental relaxation curves for 25 mM Ala–Gly–Gly dissolved in D_2_O solution acquired at 9.4 T (^1^H nuclear Larmor frequency = 400 MHz) and 298 K with two transients per data point. A delay of 230 s was used between successive transients. Samples were prepared by saturating the D_2_O solution with the following gases (O_2_ weight percentage, wt%) for 180 s prior to measurements of the singlet lifetime: black: O_2_ (>99.99); grey: compressed air (∼20.95); blue: N_2_ (<0.01). The decay profiles were obtained by implementing the SLIC pulse sequence described in Fig. 2. Singlet lifetimes (*T*_S_): black filled circles: 7.0 ± 0.1 s; grey filled triangles: 19.1 ± 0.6 s; blue filled squares: 45.6 ± 0.9 s. All signal intensities were normalized to the first data point. The fitted curves have a single exponential form.

Single exponential relaxation curves illustrating the decay of nuclear singlet order after bubbling the solution with nitrogen, compressed air and oxygen gases for 180 s are shown in Fig. 3. Singlet lifetimes *T*_S_ range from 7.0 s (O_2_ gas) to 45.6 s (N_2_ gas). Longitudinal relaxation time constants and ratios of *T*_S_ to *T*_1_ are given in Table 1.

**Table 1:** Relaxation time constants for 25 mM Ala–Gly–Gly dissolved in D_2_O solution acquired at 9.4 T (^1^H nuclear Larmor frequency = 400 MHz) and 298 K, for a range of O_2_ gas weight percentages (wt%). Sample conditions correspond to those described in Fig. 3

O_2_ wt%	Gas	*T*_S_/s	*T*_1_/s	*T*_S_/*T*_1_
>99.99	O_2_	7.0 ± 0.1	1.06 ± 0.02	6.6 ± 0.1
∼20.95	Air	19.1 ± 0.6	1.46 ± 0.02	13.1 ± 0.6
<0.01	N_2_	45.6 ± 0.9	1.60 ± 0.08	28.5 ± 0.9

The singlet relaxation time constants plateau with an increasing gas bubbling time, as demonstrated in Fig. S1.† A plateau of the singlet relaxation time is reached at 180 s, and implies saturation of the gases in solution since no further extension/depletion of the singlet lifetime is observed, see the ESI† for details.

Henry's law empirically relates the partial pressure of a gas to the dissolved quantity in solution, for low partial pressures and gas concentrations.^63^ The molar concentration of gas dissolved in solution is given by:1
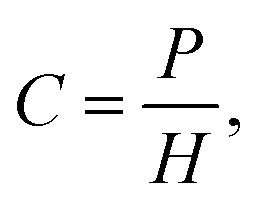
where *C* is the molar gas concentration of the solution, *P* is the partial pressure of the gas, and *H* is Henry's constant. The value of Henry's constant for O_2_ dissolved in D_2_O solvent at atmospheric pressure and 298 K is 756.7 at mL mol^−1^.^63^ Henry's law can be used to convert a wt% of oxygen gas to a molar concentration in solution. Using eqn (1), the partial pressure of oxygen gas under atmospheric conditions (*P* = 0.20948 atm), and Henry's constant for D_2_O solution, the molar concentration of oxygen dissolved in D_2_O solvent at atmospheric pressure and 298 K is determined to be: *C* = 0.2768 mM. This concentration is used to calculate the amount of oxygen in solution for each N_2_ : O_2_ gas mixture, assuming complete saturation of the gas mixtures in solution after sample bubbling for 180 s. Small deviations in sample temperature, induced by the solution bubbling process, were ignored in the estimation of dissolved oxygen concentrations.

**Fig. 4:**
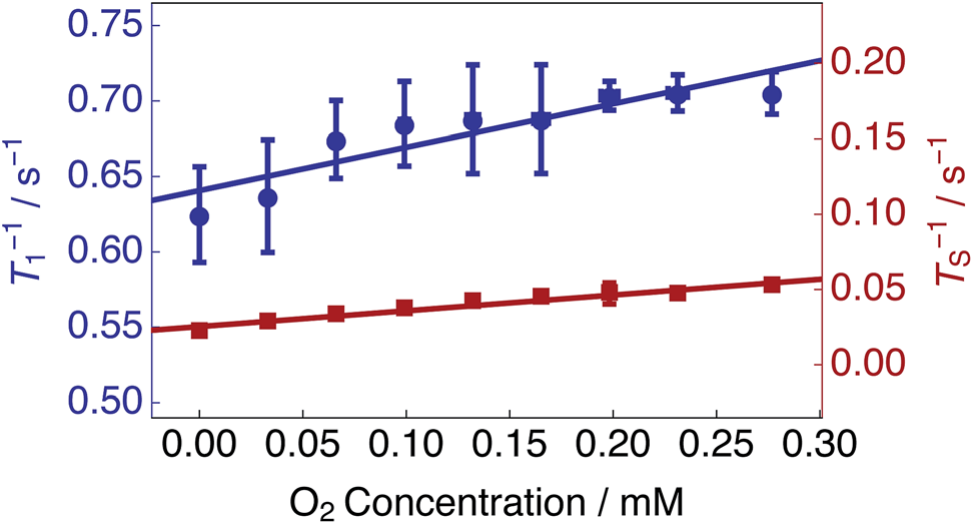
Singlet *T*_S_^−1^ (right hand axis) and longitudinal *T*_1_^−1^ (left hand axis) relaxation rate constants for 25 mM Ala–Gly–Gly dissolved in D_2_O solution acquired at 9.4 T (^1^H nuclear Larmor frequency = 400 MHz) and 298 K as a function of the O_2_ concentration in solution. Singlet lifetimes *T*_S_ (red filled squares) were estimated by using the SLIC pulse sequence described in Fig. 2. Longitudinal lifetimes *T*_1_ (blue filled circles) were measured by using the inversion-recovery pulse sequence. Samples were prepared by saturating the D_2_O solution with known ratios of O_2_ and N_2_ gas, ranging from 0–20.95% O_2_ weight percentage (wt%), for 180 s prior to measurements of the singlet lifetime. The relaxation data were fitted with a straight line function including a non-zero intercept: *T*_i_^−1^(*C*) = *r*_i_ × *C* + *T*_i_^−1^(0). Best fit values: singlet relaxation (red line): *r*_S_ = 0.105 mM^−1^ s^−1^; *T*_S_^−1^(0) = 0.025 s^−1^; longitudinal relaxation (blue line): *r*_1_ = 0.287 mM^−1^ s^−1^; *T*_1_^−1^(0) = 0.641 s^−1^. The horizontal error bars are smaller than the data points.

Estimated singlet (*T*_S_^−1^) and longitudinal (*T*_1_^−1^) relaxation rate constants as a function of the solution oxygen concentration are presented in Fig. 4 (see Methods). *T*_S_^−1^ and *T*_1_^−1^ increase linearly as a function of the dissolved oxygen concentration. A linear relationship has been described previously for singlet and longitudinal relaxation resulting from paramagnetic transition metal ions in solution.^48^

The experimental data were fit with relationships of the kind: *T*_i_^−1^(*C*) = *r*_i_ × *C* + *T*_i_^−1^(0); where *T*_i_^−1^(*C*) are the relaxation rate constants, *r*_i_ are the experimental relaxivities, *C* is the concentration of oxygen in solution (estimated by using eqn (1)), and *T*_i_^−1^(0) denotes the relaxation rate constants of an oxygen-free sample. The relaxivities for singlet *r*_S_ and longitudinal *r*_1_ relaxation were determined from the slopes of a straight line fit to the experimental data (over the range of dissolved O_2_ concentrations shown in Fig. 4) to be: *r*_S_ = 0.105 mM^−1^ s^−1^; *r*_1_ = 0.287 mM^−1^ s^−1^. The singlet relaxivity is a factor of ∼2.7 smaller than the relaxivity for longitudinal relaxation. These results indicate that singlet order is less sensitive than conventional magnetization to relaxation induced by paramagnetic oxygen dissolved in solution.

An oxygen concentration of ∼0 mM reveals the effects of additional contributions to longitudinal and singlet relaxation. The motional modulation of the in pair dipole–dipole coupling between the glycyl protons provides a dominant source of relaxation for longitudinal magnetization, but is absent for singlet order.^52,64^ For the case of a solution saturated with N_2_ gas, an impressive relaxation time ratio *T*_S_/*T*_1_ = 28.5 is observed, see Table 1.

## 4 Discussion

The ratio *r*_S_/*r*_1_ can be understood by employing a model in which the protons of the glycine residue experience a correlated fluctuating field generated by nearby paramagnetic oxygen species dissolved in solution.^65–68^ In the extreme-narrowing limit, relaxation rate expressions for *T*_S_^−1^ and *T*_1_^−1^ are:

2 *T*_S_^−1^ = 2*γ*_H_^2^(*B*_1_^2^ + *B*_2_^2^−2*κ*_HH_*B*_1_*B*_2_)*τ*_C_,

3 *T*_1_^−1^ = *γ*_H_^2^(*B*_1_^2^ + *B*_2_^2^)*τ*_C_,

where *γ*_H_ is the proton gyromagnetic ratio, *B*_1_ and *B*_2_ are the root-mean-square amplitudes of the correlated fluctuating fields at the proton sites, *τ*_C_ is the rotational correlation time for the overall tumbling motion as the molecule reorients in solution, and *κ*_HH_ is a proportionality constant which describes the extent of the fluctuating field correlations. In the case of strong relaxivities or high oxygen concentration, *i.e. r*_i_*C* > *T*_i_^−1^(0), the ratio of the singlet and longitudinal relaxation rates is highly dependent upon the field correlation factor *κ*_HH_:4
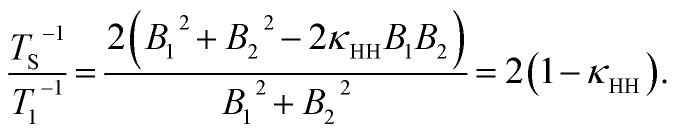


The assumption *B*_1_ = *B*_2_ has been included in the final step. Using eqn (4), and the ratio of *r*_S_/*r*_1_ = (2.7)^−1^, a correlation factor *κ*_HH_ = 0.82 is obtained, *i.e.* a strong correlation of the fluctuating fields at the nuclear sites. Similar values of *κ*_HH_ were found for the case of paramagnetic lanthanide salts dissolved in solution.^48^ The value of *κ*_HH_ implies that the two protons are somewhat close in space, relative to the proton–oxygen separations, and that there is likely to be a narrow subtended angle between the singlet nuclei and the paramagnetic oxygen species. Therefore, oxygen species must approach relatively closely to the spin-1/2 pair in order to significantly attenuate the singlet relaxation time. Further exploration of this behaviour is beyond the scope of this paper but estimates of the singlet relaxation rate induced by the presence of paramagnetic oxygen species dissolved in solution have previously been addressed in the literature,^42,50^ and could be further explored by deploying molecular dynamics simulations.^47^

**Fig. 5:**
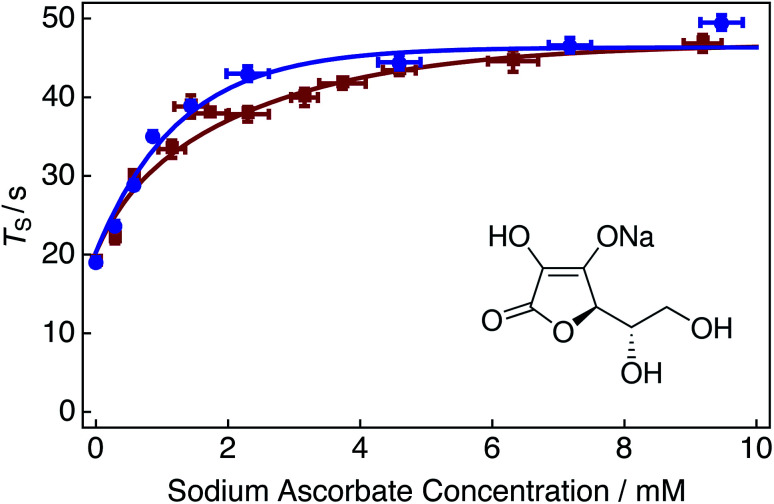
Singlet relaxation time constants *T*_S_ of 25 mM Ala–Gly–Gly dissolved in D_2_O solution acquired at 9.4 T (^1^H nuclear Larmor frequency = 400 MHz) and 298 K as a function of the sodium ascorbate concentration in solution. Singlet lifetimes were estimated by using the SLIC pulse sequence described in Fig. 2. Samples were initially prepared by saturating the D_2_O solution with compressed air gas (∼20.95% O_2_ weight percentage, wt%) for 180 s. Sodium ascorbate was subsequently added step-wise to the solution. The delay between sample preparation and commencing singlet lifetime measurements was 15 minutes in each case. Red filled squares: no tap water added to solution; blue filled circles: 2 μL tap water added to solution. The fitted lines are intended to guide the eye. Inset: molecular structure of the oxygen radical quenching agent sodium ascorbate.

Oxygen induced relaxation can be attenuated by quenching dioxygen, hydroperoxide and superoxide radical species dissolved in solution through chemical interaction with ascorbate forming diamagnetic complexes (Fig. 5, inset).^69^ Ascorbate has previously been used in LLS experiments to reduce the relaxation effects of paramagnetic agents,^48^ and to scavenge radicals in dissolution-dynamic nuclear polarization (dDNP) studies in the pursuit of minimizing polarization losses during low field sample transfer.^70^

D_2_O solutions were subjected to incremental additions of sodium ascorbate. After the addition of sodium ascorbate, samples were stored at 298 K for 15 minutes to allow the sample temperature to stabilize and for shifts in ^1^H resonance positions to settle. The experimental ^1^H NMR resonances of sodium ascorbate did not overlap with the relevant spectral region of Ala–Gly–Gly.

Fig. 5 shows singlet relaxation time constants *T*_S_ as a function of the sodium ascorbate concentration in solution. The red curve demonstrates that singlet lifetimes increase with increasing sodium ascorbate concentration. A plateau is reached at a concentration of ∼10 mM, despite further addition of the chelating agent. This plateau is most likely due to the decreased availability of oxygen radicals in solution, leading to the decreased efficacy of the oxidation of sodium ascorbate (since encounters between sodium ascorbate and free oxygen radicals are of lower probability). The singlet lifetime recorded in the absence of sodium ascorbate (Fig. 5, leftmost data point) is given in Table 1 (compressed air). The singlet lifetime measured in the presence of ∼12 mM sodium ascorbate (Fig. 5, rightmost red data point) corresponds to a value of *T*_S_ = 46.8 ± 1.1 s. The addition of sodium ascorbate clearly reduces the relaxation effects induced by dissolved radical oxygen species in solution. This value is in good agreement with that achieved by saturating the solution by bubbling with nitrogen gas (Fig. 3). This result is expected since both methods eliminate the source of paramagnetic oxygen induced relaxation. Bubbling solutions with N_2_ gas may be preferred to avoid sample contamination and overlapping spectral regions which may be problematic for ^1^H NMR measurements.

It is interesting to note that a 1 : 1 ratio of sodium ascorbate to O_2_, *i.e.* ∼0.28 mM of sodium ascorbate, is insufficient as to quench the effects of oxygen induced relaxation in solution. In reality, a relatively large amount of sodium ascorbate (∼10 mM) is required to sufficiently suppress such effects. It is possible that the initial 15 minute waiting period is too short as to allow the reaction of oxygen and sodium ascorbate to fully complete. The kinetics of this reaction have not been investigated further.

Ascorbate can act as a bidentate ligand which has been shown to chelate to metal ions.^71^ Transition metal ions can catalyse the oxidation of ascorbate (quenching of radical oxygen) through the formation of intermediary metal ion–ascorbate–dioxygen complexes.^71^ This mechanism has been investigated for both iron and copper ions. It has been shown that ferric and cupric ions in solution result in the catalysis of ascorbate oxidation. Fe(iii) and Cu(ii) ions are usually found at low concentrations in regular tap water. Compositional data, published by Southern Water UK, the regional water supplier, states the iron and copper ion content of tap water to be 18.55 and 0.078 ppb, respectively.^72^

The blue curve in Fig. 5 shows singlet lifetimes for a range of sodium ascorbate concentrations in the case that a 2 μL aliquot of tap water was added to the D_2_O solution. Relaxation from paramagnetic iron and copper centers was assumed to be negligible. Following the addition of tap water, the maximum value of *T*_S_ is reached at lower sodium ascorbate concentrations (∼5 mM). The reaction catalysis is demonstrated by the sharper initial increase of singlet lifetimes at reduced sodium ascorbate concentrations.

## 5 Conclusions

In Conclusion, the singlet lifetime of the proton pair in the glycine-terminal residue of the polypeptide Ala–Gly–Gly has been investigated as a function of the dissolved oxygen concentration in solution. Singlet relaxation rate constants were found to be linearly dependent on the quantity of oxygen in solution. The ratio of the relaxivities for singlet and longitudinal relaxation was found to be ∼(2.7)^−1^. Singlet and longitudinal relaxivities were described using a model of correlated fluctuating fields, with a correlation factor *κ*_HH_ = 0.82. It should also be noted that alternative mixtures of molecules and solvents will have unique correlation factors *κ*_HH_ dependent on the closest approach of paramagnetic oxygen to the target nuclei, the solubility of oxygen gas in solution, and the sample temperature. Nevertheless, the method presented here provides a relatively simple approach to establish known concentrations of oxygen in solution and for calibrating the extent of relaxation caused by dissolved oxygen, under suitable circumstances. The singlet lifetime was investigated in the presence of the radical scavenging salt sodium ascorbate. It was observed that relatively large concentrations of sodium ascorbate were required to quench relaxation effects from dissolved oxygen in solution. The presence of tap water was found to catalyse the oxidation of sodium ascorbate in solution.

## Conflicts of interest

There are no conflicts of interest to declare.

## Supplementary Material

RA-009-C9RA03748A-s001
