# Supplementary material for: Experimental evidence for the role of paramagnetic oxygen concentration on the decay of long-lived nuclear spin order
Source: RSC Adv. 2019 Jul 29;9(40):23418–24. doi: 10.1039/c9ra03748a (PMC9067289; doi:10.1039/c9ra03748a)
Supplement: RA-009-C9RA03748A-s001 [file RA-009-C9RA03748A-s001.pdf]

Electronic Supplementary Material (ESI) for RSC Advances.
This journal is © The Royal Society of Chemistry 2019

Supplementary Information

Experimental evidence for the role of paramagnetic oxygen concentration on the decay of long-lived nuclear spin order

Bryan Erriah[a] and Stuart J. Elliott[a*†]

[a]*School of Chemistry, University of Southampton, Southampton SO17 1BJ, United Kingdom*
[*]*Corresponding Author: stuart-james.elliott@univ-lyon1.fr*
[†]*Current Address: Centre de Résonance Magnétique Nucléaire à Très Hauts Champs - FRE 2034 Université de Lyon / CNRS / Université Claude Bernard Lyon 1 / ENS de Lyon, 5 Rue de la Doua, 69100 Villeurbanne, Lyon, France*

Contents

1 Singlet Lifetime vs. Solution Bubbling Time 3

1. Singlet Lifetime vs. Solution Bubbling Time

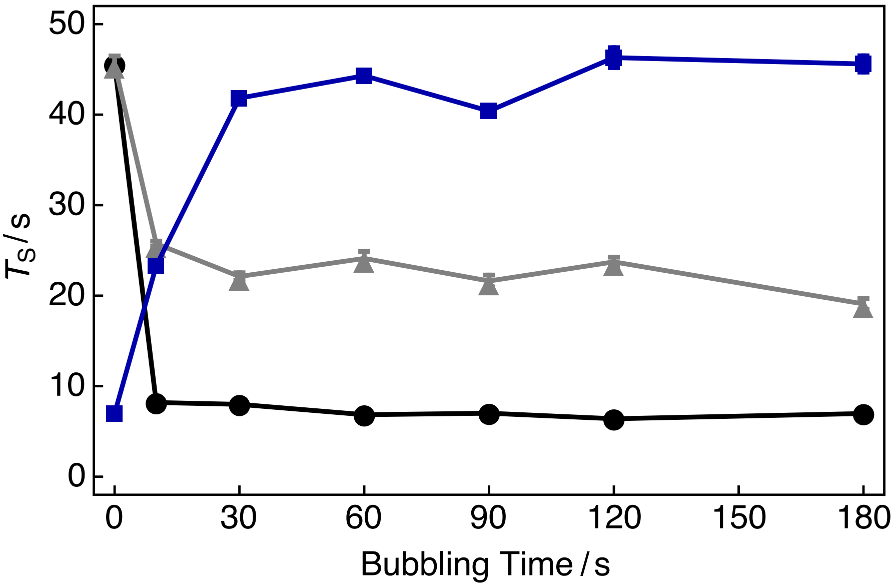

Figure S1: Singlet relaxation time constants T_S for 25 mM Ala-Gly-Gly dissolved in D_2O solution acquired at 9.4 T (^1H nuclear Larmor frequency = 400 MHz) and 298 K as a function of the gas bubbling time. Singlet lifetimes were estimated by using the SLIC pulse sequence described in Figure 2 of the main text. Starting conditions (leftmost data points): Samples were initially prepared by saturating the D_2O solution with the following gases (O_2 weight percentage, wt.%) for 180 s: Black filled circles: O_2 (>99.99); Grey filled triangles: N_2 (<0.01); Blue filled squares: N_2 (<0.01). Saturation experiments: Samples were subsequently bubbled with the following gases (O_2 weight percentage, wt. %) for an incremented gas bubbling time before measurements of the singlet lifetime: Black filled circles: O_2 (>99.99); Grey filled triangles: compressed air (~20.95); Blue filled squares: N_2 (<0.01).

The singlet relaxation time constants plateau with an increasing gas bubbling time, as demonstrated in Figure S2. The blue and black lines indicate samples which have been bubbled with nitrogen and oxygen gases, respectively. A plateau of the singlet relaxation time is reached at 180 s (blue curve), and implies saturation of the N_2 gas in solution since no further extension of the singlet lifetime is observed. Similarly, the plateau of the black curve illustrates the saturation of O_2 gas in solution. The two gases saturate the D_2O solution on a similar timescale. The grey line shows the behaviour of a solution treated with compressed air, and demonstrates the fluctuations associated with experimental error. It is worth noting that other combinations of molecules and solvents will have different saturation timescales based on oxygen solubility in solution and sample temperature.
